# Impact of Lactation Support Program on Initiation of Breastfeeding in Term Infants

**DOI:** 10.31372/20190403.1059

**Published:** 2019

**Authors:** Binu Ninan, Umamaheswari Balakrishnan, Asiff Mohamed, Munusamy Manjula, Thangaraj Abiramalatha, Ashok Chandrasekaran, Prakash Amboiram

**Affiliations:** Sri Ramachandra Institute of Higher Education and Research, Chennai, India

**Keywords:** lactation counselors, improved breastfeeding rate, early initiation, lactation support program

## Abstract

*Purpose*: Early initiation of breastfeeding (EIBF) significantly decreases neonatal mortality and improves exclusive breastfeeding. The objective of the present study was to assess the effect of lactation support program (LSP) on early initiation of breastfeeding (BF) among term well infants. *Methods*: A “before-and-after” design was used to study the effect of the LSP on EIBF at a tertiary care institute in India over a period of two and half years. EIBF was defined as BF initiated <1 hour in vaginal delivery (VD) and <2 hours in cesarean section (CS). Impact of LSP was assessed by comparing baseline data (control group) with data after initiation of LSP (study group). Even after 1 year of initiation of LSP, EIBF in CS remained low, hence a hospital policy was implemented to alter a modifiable factor to promote EIBF in CS. Data of the study group was analyzed over two time periods, as study group A (prior to implementation of hospital policy) and study group B (following the commencement of hospital policy). *Results*: A total of 2,769 postnatal mothers were included for the study with 537 in the control group, 1,157 in study group A, and 1,075 in study group B. In VD, EIBF rate increased significantly from 92.6% at baseline to 99.8% and 99.6%, in study group A and study group B, respectively (*p* value < 0.001). In CS, EIBF rate increased from 0.4% at baseline to 1.9% and 92.7% in study group A and study group B, respectively (*p* < 0.001). The time of initiation of BF reduced from 1.3 (0.9) to 0.7 (0.3) hours in VD and from 4.2 (0.71) to 1.8 (0.66) hours in CS with both having a *p* value of < 0.001. *Conclusion*: Lactation support program is a simple but effective way of implementing appropriate steps towards promotion of exclusive BF.

## Background

Breastfeeding (BF) is recognized as the single most important measure to reduce under-five mortality (Jones et al., 2003). Early initiation of BF (EIBF) significantly decreases neonatal mortality and improves the rate of exclusive BF (Khan, Vesel, Bahl, & Martines, 2015; A. Patel, Banerjee, & Kaletwad, 2013). World Health Organization recommends EIBF within one hour of birth (“WHO ∣ Early initiation of breastfeeding to promote exclusive breastfeeding,” n.d.). However, despite the many initiatives taken at the national and international level, the rate of EIBF remains unacceptably low. As per the data of United Nations International Children’s Emergency Fund (UNICEF) (2016) the rate of EIBF was only 44% in the world, 39% in South Asia and 41% in India (Watkins, 2016). Studies have shown that BF education and support improve BF outcomes (Haroon, Das, Salam, Imdad, & Bhutta, 2013; Imdad, Yakoob, & Bhutta, 2011; McFadden et al., 2017; S. Patel & Patel, 2016). Lack of awareness and gaps in the mothers’ knowledge on BF are some of the important factors that contribute to low BF rates. Educational and BF promotional programs could bridge this gap. In a systematic review by Haroon et al., combined individual and group counseling had a greater effect of BF especially in developing countries (Haroon et al., 2013). Though there are several processes involved in establishing exclusive BF, the initial component lies during the immediate postnatal period, where the mother–infant dyad stays in the hospital. Thus we intended to promote early initiation of BF in well term infants during their stay in our hospital.

The selected hospital is a tertiary care institute with outpatient facility, inpatient facility, and intensive care units for both mother and children with an average of 1,500 live births per year. The hospital caters middle-income and high-income group, and high-risk as well as low-risk deliveries. Before the initiation of this study, there were no assigned personnel or support program for guidance of the mothers who had well neonate. Hence we identified the need for a support program and initiated lactation support program (LSP) in our hospital for enabling exclusive breastfeeding. Further, we aimed to study the effect of LSP on the early initiation of BF in well term infants.

## Methods

The study was done in a tertiary care institute in India from July 2013 to December 2015. This was a before- after study design, wherein baseline data on EIBF obtained before the commencement of LSP was compared with data on EIBF obtained after initiation of LSP. Newborn infants born intramural at or above 37 weeks gestation who were well, were included in the study. We included babies irrespective of their growth status [appropriate for gestational age (AGA), small for gestational age (SGA), or large for gestational age (LGA)] provided they reminded well and were able to feed. We excluded neonates anticipated to have problems with BF due to congenital anomalies or being transferred to neonatal unit for other medical reasons. Since the study was a part of initiatives taken to improve the quality of care delivered to patients in the institute, we did not obtain approval from institutional review board for the study.

The lactation support program (LSP) was initiated from January 2014. The components of LSP were antenatal counseling, postnatal assessment, continuing support during follow up, constant surveillance, internal audits, and regular inter-professional team meets. The stakeholders of LSP were lactation consultant (LC), consultant neonatologists (CN), pediatricians, and obstetricians, though the entire program was revolving around the activities of LC. We had two trained and dedicated nurses as lactation consultants (LC), who worked for 8 hours a day on all working days. They had undergone “Infant and Young Child feeding Counseling specialist course” and identified themselves as key persons involved in supporting lactation.

Antenatal counseling sessions were conducted by LC twice a week for 20 minutes duration in outpatient department for pregnant women during the third trimester of pregnancy in small groups of 4–8. Information was given regarding the importance of EIBF, continuation of exclusive BF till 6 months, advantages of BF, disadvantages and possible adverse effects of cow’s milk, pre-lacteal feeding or formula milk feeding, addressing misconception about colostrum and the technique of BF with audio-visual aids (PowerPoint presentation and video clips). The LCs also conducted education sessions once a week for the nurses in postnatal wards regarding the importance of EIBF. LC assessed all mother–infant dyad every day from delivery till discharge for BF and helped with issues of BF. LC also provided additional help to the nursing mothers on a need basis. The functioning of LC was closely integrated with the consultant neonatologist (CN). CN carried out postnatal ward rounds to examine neonates everyday while the LC reported on the feeding status of all neonates in the PN ward. Need for additional support from LC to initiate BF was also assessed by CN through conversations with the mothers. Moreover, as CN attended all the deliveries, both VD and CS it was easy to establish EIBF. In VD, once the infant was handed over to the mother after initial assessment, she was encouraged by CN to initiate BF early. In CS, non-drug order was placed in the infant’s case sheet so that the neonatal nurses could take the infant to BF. The next component namely “continuing support during follow up” was provided by LC in OP department during the neonatal follow up and subsequent follow up during infancy at the time of immunization. On these occasions, the importance of exclusive BF was re-emphasized and had been offered help in lactation if needed. The functioning of LSP was reviewed on regular basis at 6 monthly interval by the consultant in-charge of the neonatal services along with other CN wherein the work of LC was appraised and strategies to improve all components of LSP were discussed along with review of the data. Inter-professional team meets were conducted every year wherein the data collected by LC was reviewed.

**Figure 1:**
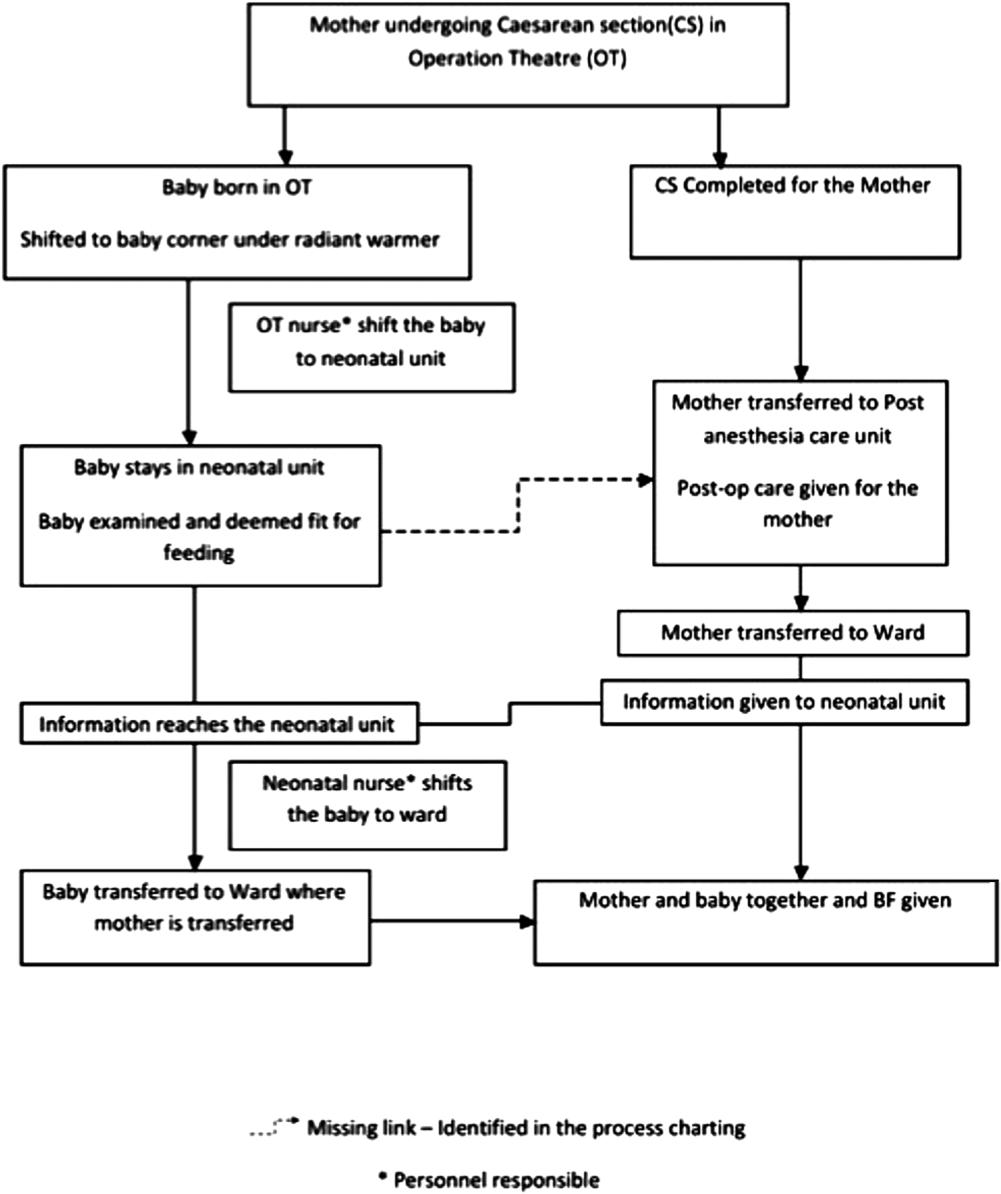
Process chart on handling babies born in operation theatre.

An interim audit done in the month of January 2015 reviewing the one-year data after initiation of LSP, showed that the rate of EIBF was considerably low in babies born by cesarean section (CS) despite the measures taken. The entire process involved in EIBF among babies born through CS was reviewed and Figure 1 depicts the process flow chart. As a unit policy, all babies born by CS were kept in nursery till the mother was shifted to postnatal ward. BF was initiated only once the mother had been shifted to postnatal ward, in other words, BF was not initiated while the mother remained in Post Anesthesia Care Unit (PACU). This missing link was identified and hence, a hospital policy of initiating BF in PACU within the operation theatre (OT) premises was implemented in March 2015 to reinforce EIBF. This policy change involved interaction of neonatologist with anesthetists and obstetricians, and also training and education of nurses in PACU and neonatal unit. The neonatal nurse taking care of the baby in the nursery was given the responsibility to take the baby to the mother and initiate BF in PACU within 2 hours of life.

The LC and the consultant neonatologist on postnatal ward rounds checked the time of initiation of BF for every baby. On circumstances when EIBF was not initiated within the stipulated time or delayed, feedback about the same was given to the nurse responsible for the mother–infant dyad, the reasons behind was analyzed, the importance of EIBF, and exclusive BF were re-emphasized. Data collected by LC included the demographic and clinical data of well infants, data on time of initiation of BF, EIBF status, and discharged on exclusive BF or not. These were collected during the hospital stay of mother–infant dyad. The average length of stay of infant born through VD and CS were 2 days and 5 days, respectively.

EIBF was defined as initiation of BF within 2 hours in infants born through CS and within 1 hour in infants born through VD in this study. Our unit protocol mandates the documentation of the time of initiation of BF and status of BF at discharge in the medical case record by the nurses. Control group comprised of baseline data including EIBF, relevant demographic and clinical data collected retrospectively from the medical case records, for a period of 6 months prior to initiation of LSP (July–December 2013). Data of the study group was analyzed over two time periods, as study group A (prior to implementation of hospital policy) and study group B (following the commencement of hospital policy). Study group A comprised of infants enrolled from January 2014 to February 2015 and study group B comprised of infants enrolled from March 2015 to December 2015. Outcome assessed were the rate of EIBF and time to initiate BF.

### Statistical Methods

Data were represented as mean and standard deviation (SD), or number and percentage as appropriate. Chi-square was used for categorical data and ANOVA was used to compare continuous normal data. The association between exclusive BF at discharge (dependent variable) and other factors including primigravida, CS as mode of delivery, and EIBF (independent variables) was analyzed by multiple logistic regression analysis. All statistical analyses were done using SPSS 16.0. A *p*-value less than 0.05 was considered statistically significant.

## Results

There were a total of 4,085 women who delivered during this period, out of which 2,769 were included for the study. We had 537 in the control group, 1,157 in study group A, and 1,075 in study group B. The baseline characteristics of the mother and the baby were comparable between the control and the study groups (Table 1).

**Table 1:** Baseline Characteristics

	Controls	Group A	Group B
	(*n* = 537)	(*n* = 1,157)	(*n* = 1,075)
Maternal age, mean (SD)	27.9 (3.7)	27.8 (3.8)	28.2 (3.9)
Primigravida; *n* (%)	299 (56)	666 (58)	537 (50)
Mode of delivery; *n* (%)			
Normal	226 (42)	492 (43)	480 (45)
Instrumental vaginal	15 (3)	28 (2)	39 (4)
Elective cesarean	139 (26)	258 (22)	251 (23)
Emergency cesarean	157 (29)	379 (33)	305 (28)

**Figure 2:**
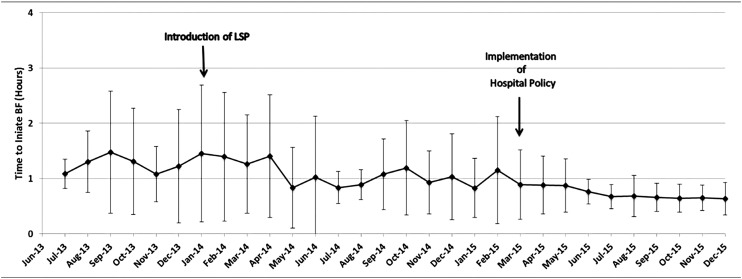
Time to initiate BF in vaginal delivery (mean ± SD).

**Figure 3:**
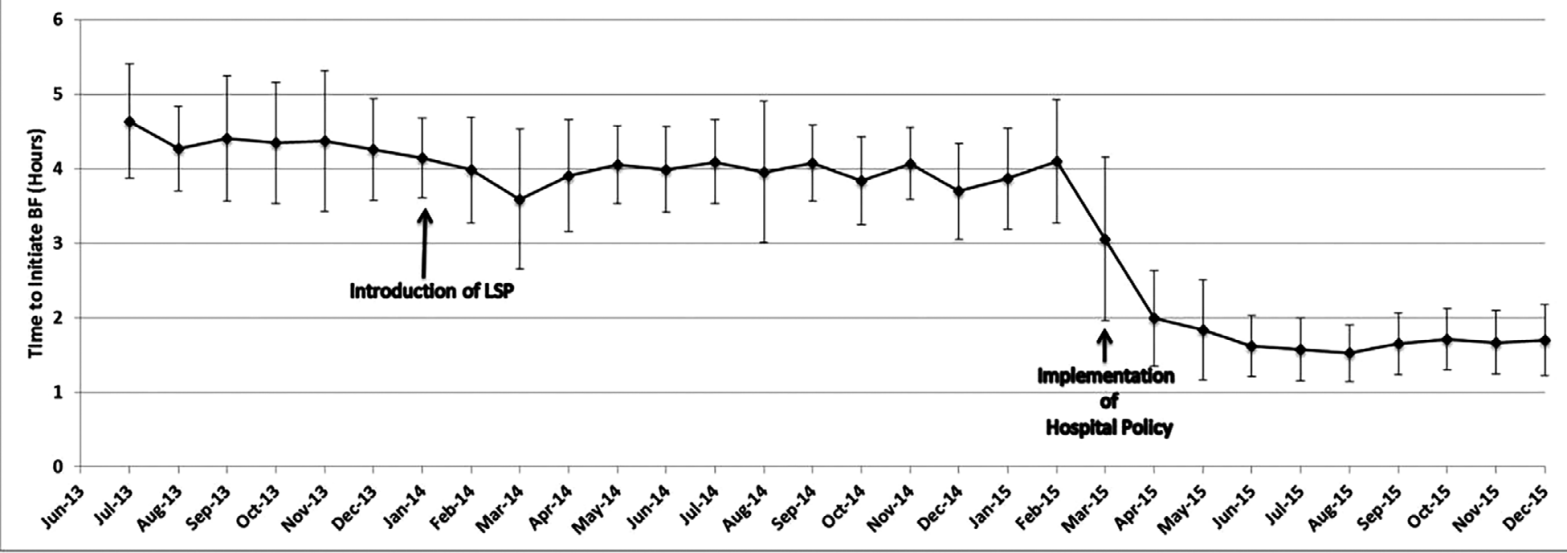
Time to initiate BF in cesarean section (mean ± SD).

For babies born by VD, there was a significant increase in the rate of initiation of BF within 1 hour from 92.6% in control group to 99.8% and 99.6%, in study group A and group B, respectively (*p* value <0.001) (Table 2). Among infants delivered by CS, initiation of BF within 2 hours increased marginally from 0.4% in control group to 1.9% in study group A, followed by a remarkable increase to 92.7% in study group B (*p* value <0.001). The rates of EIBF did not vary among elective or emergency CS. Initiation of BF within 1 hour in CS had increased from 0.4% in control group and 0.3% in study group A to 16.5% in study group B. There was a significant improvement in the time to initiate BF in both VD and CS which had been sustainable for more than 6 months (Figures 2 and 3 and Table 2). Exclusive BF at the time of discharge during control period was 97.1% and it increased to 98.9% after initiation of LSP (*p* < 0.01). Among the total of 2,769 postnatal mothers, 39 (1.4%) did not exclusively BF at discharge, out of which 10 (0.7%) had VD and 29 (1.97%) had CS. There were no significant differences between elective and emergency CS (1.7% vs. 2.2%). When we analyzed the factors for exclusive BF at discharge by multiple logistic regression analysis, CS was independently associated with exclusive BF (*p* < 0.05) whereas EIBF and primigravida were not associated.

**Table 2:** Initiation of Breastfeeding after Delivery

Vaginal delivery				
	Controls	Group A	Group B	*p* value
	(*n* = 259)	(*n* = 520)	(*n* = 525)	
Initiation of breastfeeding within 1 hour; *n* (%)	240 (92.6)	519 (99.8)	518 (99.6)	<0.001∗
Time to initiate breast feeding (hours); mean (SD)	1.3 (0.9)	0.8 (0.3)	0.7 (0.3)	<0.001#
Time to initiate breast feeding (hours) 95% CI	1.2–1.4	0.7–0.8	0.6–0.7	
Cesarean section				
	Controls	Group A	Group B	*p* value
	(*n* = 278)	(*n* = 637)	(*n* = 550)	
Initiation of breastfeeding within 2 hours; *n* (%)	1 (0.4)	12 (1.9)	510 (92.7)	<0.001∗
Time to initiate breast feeding (hours): mean (SD)	4.2 (0.71)	3.9 (0.63)	1.8 (0.66)	<0.001#
Time to initiate breast feeding (hours) 95% CI	4.2–4.4	3.8–3.9	1.7–1.8	

∗Chi-square test; #ANOVA test.

## Discussion

Our study shows that EIBF is achievable with support program for lactation. The authority to implement new hospital policy is noteworthy activity of LSP. Collaboration across different specialties, breaking the barriers, and integrating LC in routine work schedule are some of the key features of LSP. Team effort, dedicated LC, rigorous hospital policy, and commitment of healthcare workers have contributed for successful EIBF. A recent qualitative study has shown that LC perceived lack of coordination among professionals as one of the barriers for providing BF support (Anstey et al., 2018). However LSP could help to liaise across to help break the barriers and improve the inter-professional interaction among healthcare providers. One of the targets of Every Newborn Action Plan (ENAP) is to achieve EIBF in 90% of deliveries, which we could achieve in our study in VD. In babies delivered by CS, BF could be initiated within 2 hours by the policy of initiating BF in PACU.

Many cultural and socio-demographic factors could hinder EIBF and colostrum feeding (A. Patel et al., 2013; Sharma & Byrne, 2016). The barriers for EIBF include traditional belief that colostrum is harmful to the baby, pre-lacteal feeding, priests’ advice, perceived milk insufficiency, and not involving the mothers in decision making (Sharma & Byrne, 2016). Lack of correct information to mothers is a major barrier. Hence, addressing all misconceptions of the mothers regarding colostrum, pre-lacteal feed, educating her about the importance of EIBF, and providing motivation and support improve the rate of EIBF as shown in our study.

A recent systematic review found that measures shown to improve EIBF include baby friendly hospital initiative (BFHI), BF support at hospital and community level, and counseling and education of antenatal mothers (Rollins et al., 2016). Our institution despite being a BFHI hospital, the baseline rate of early initiation was low. With implementation of LSP and commitment of healthcare workers, we could improve the rate of EIBF even in babies born through CS in our study. Perioperative lactation program has been implemented with success in a U.S.-based hospital to address the comprehensive care of lactating patients in anesthesiology set-up, but this study addressed the group of mothers who have been already lactating mothers unlike our study (Rieth, Barnett, & Simon, 2018).

EIBF is one of the core indicators for assessing infant feeding practices (WHO_CDD_SER_91.14.pdf, n.d.). A recent survey conducted across 24 countries including India reports prevalence of EIBF ranging from 17.7% to 98.4% (average 57.6%). Twenty facilities have participated in India and the rate was 65.8%. In this survey, EIBF was significantly lower in those delivered by CS (Takahashi et al., 2017). CS has been reported to be a barrier for EIBF in a systematic review (Prior et al., 2012). In another review, though CS was not reported to be a risk factor with adequate support, yet the support was not described (Rollins et al., 2016). We could achieve EIBF in CS in majority of the infants. However, we kept a pragmatic definition of EIBF as initiating BF within 2 hours in cesarean section (CS), as it was difficult in practice to implement within 1 hour and this was what we could best achieve. After the birth of the baby through CS, completion of uterine suturing, securing homeostasis, and skin suturing took on an average 30 to 40 minutes depending on the expertise of the person conducting the CS, followed by shifting of mother to PACU from the OT in 15 minutes. Once the mother reached PACU, the call was made to neonatal unit, as the OT complex was situated in 6^th^ floor and neonatal unit in 3^rd^ floor; it took another 10 to 15 minutes to bring the neonate to PACU. These were the practical difficulty we faced in implementing within 1 hour. A study conducted in Japan to determine ideal timing taken for initiation of BF, found that initiating BF within 120 minutes have been associated with exclusive BF at 4 months (Nakao, Moji, Honda, & Oishi, 2008). In our study, the rate of EIBF within 1 hour was only 16% in CS in spite of hospital policy. In another quality initiative study to improve first hour BF, BF was initiated on OT table during CS itself (Dudeja, Sikka, Jain, Suri, & Kumar, 2018). These reinforce that we might have to adopt some other strategy to make EIBF possible within 1 hour in CS.

In spite of these variations, we had a high rate of exclusive BF at discharge. Even in the small population where exclusive BF was not done, we observed that CS was an independent factor affecting the same, after adjusting for EIBF and parity. There could be potentially other reasons in this group as CS generally carried out in high-risk population, though we did not analyze the specific reasons for initiating alternate feeds.

The present study has identified the following limitations. The data on controls was collected retrospectively. The cost effectiveness was not analyzed in this study but additional element needed to implement LSP was to allocate 2 nurses and train them to take up the role of LC. Considering the impact of LSP, it could potentially be considered as a cost effective method. We are continuing the LSP in our institute, which underscores that LSP is sustainable and the belief that it is cost-effective. Possibility of adding a component involving technology could be a way forward to improve the existing LSP. Recent studies have incorporated video-conferencing, texting, or cyber-tutor platform to improve the success of support programs (Friesen, Hormuth, Petersen, & Babbitt, 2015; Martinez-Brockman et al., 2018; Prado et al., 2013).

EIBF was possible with support program, rigorous hospital policy, and commitment of the healthcare workers. Lactation support program is a simple but effective way of implementing appropriate steps towards promotion of exclusive BF and it is also sustainable.

## Acknowledgments

Authors thank Ms Cynthia Milton for the help in language editing and formatting of the article.

## Declaration of Conflicting Interests

The authors declared no potential conflicts of interest with respect to the research, authorship, and/or publication of this article.

## Funding

This research received no specific grant from any funding agency in the public, commercial or not-for-profit sectors.
